# Supplementary material for: The cost of electrifying all households in 40 Sub-Saharan African countries by 2030
Source: Nat Commun. 2023 Aug 21;14:5066. doi: 10.1038/s41467-023-40612-3 (PMC10442407; doi:10.1038/s41467-023-40612-3)
Supplement: Supplementary file 1 — Supplementary Information [file 41467_2023_40612_MOESM1_ESM.pdf]

# Supplementary Information for

# The Cost of Electrifying All Households in 40 sub-Saharan African countries by 2030

Florian Egli[1,2], Churchill Agutu[1,3], Bjarne Steffen[4,5,6], Tobias S Schmidt[1,4,6,7]

Contains:

- Supplementary Figures S1 – S6
- Supplementary Tables S1 – S8

[1]Energy and Technology Policy Group, ETH Zurich, Zurich/Switzerland
[2]IIPP Institute for Innovation and Public Purpose, UCL, London/UK
[3]Kigali Collaborative Research Center, Kigali/Rwanda
[3]Climate Finance and Policy Group, ETH Zurich, Zurich/Switzerland
[4]Institute for Science, Technology and Policy, ETH Zurich, Zurich/Switzerland
[5]Center for Energy and Environmental Policy Research, Massachusetts Institute of Technology, Cambridge/USA
[6]Andlinger Center for Energy and the Environment, Princeton University, Princeton/USA
[7]Center for Policy Research on Energy and the Environment, Princeton University, Princeton/USA

## Additional Figures

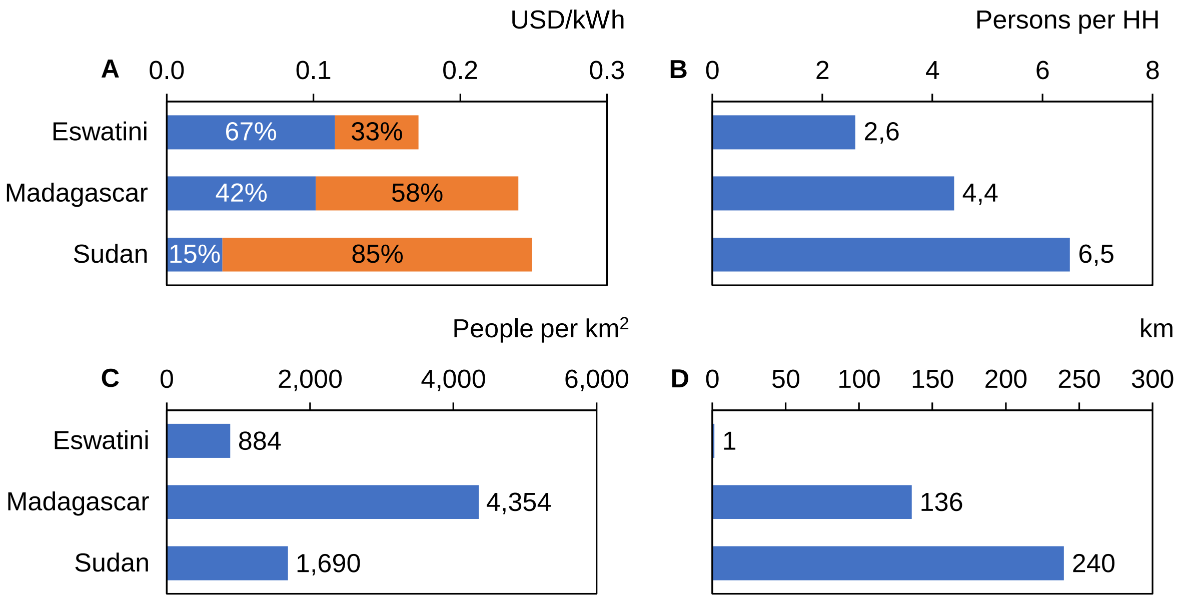


**Figure S1:** Extreme case country comparison. **A.** LCOE in USD/kWh where blue indicates the grid extension cost share and orange the SAS cost share. **B.** Average household size in person per household. **C.** Average cluster population density in people per km$^2$. Note that this shows the population density for clusters to be electrified – hence populated areas – and the average across clusters. The numbers therefore should not be compared to country-wide population density data. **D.** Average distance to closest medium voltage line in km. Numbers are always averages across areas to be electrified.

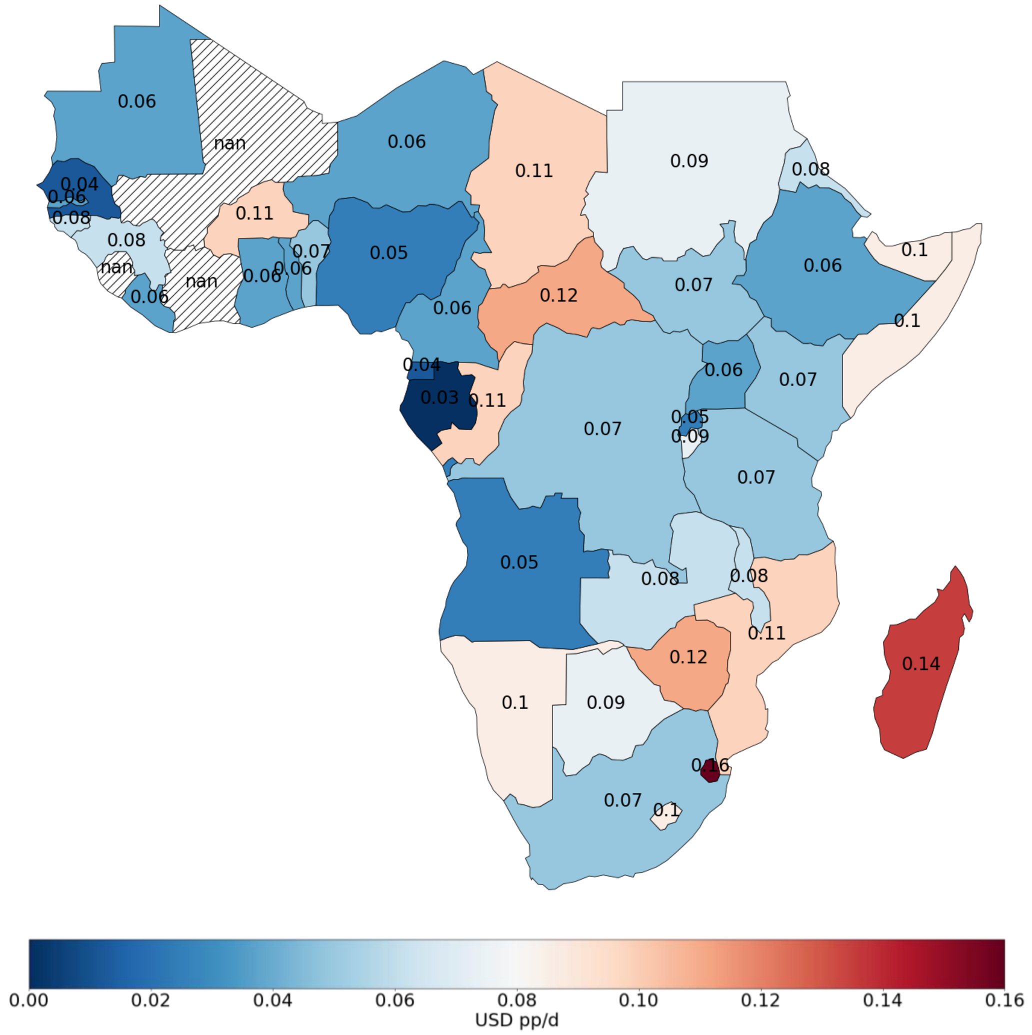


**Figure S2:** Least-cost electrification costs pp/d by country.

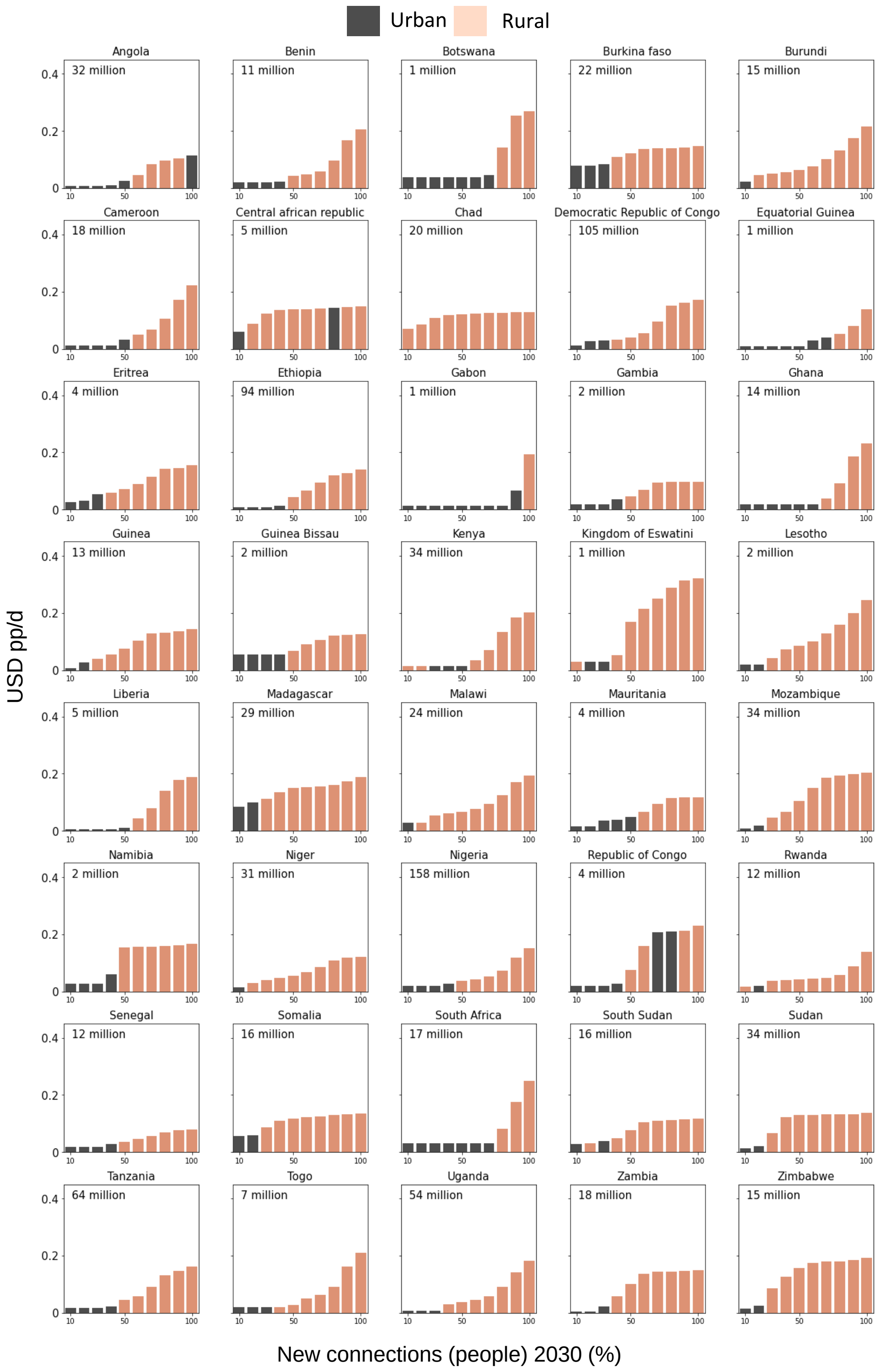


**Figure S3:** Electrification cost curves in pp/d for rural versus urban populations by country. Costs are in USD pp/d, each bar represents a decile of the population to be electrified by 2030 to reach 100% electrification. Based on an empirical threshold for urban population density by country[1], clusters are classified as urban or rural. Colours are assigned based on the classification of the majority of clusters per decile. Costs are shown for Tier 3 electrification.

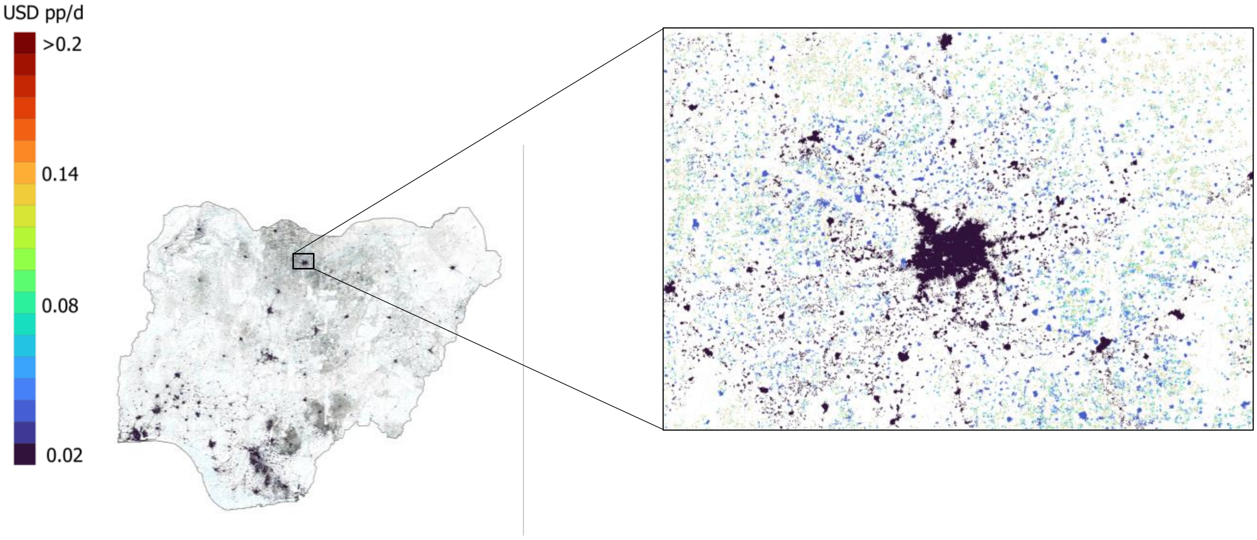


**Figure S4**: Illustration of spatially explicit cost per person per day for different clusters. The figure shows the example of Nigeria (left) and Kano city in the north of the country (inlay). The maps illustrate how costs are lowest in densely populated areas and along grid lines ("arteries" spreading from the city center). The maps are plotted using cluster data provided by the World Bank's Global Electrification Platform.

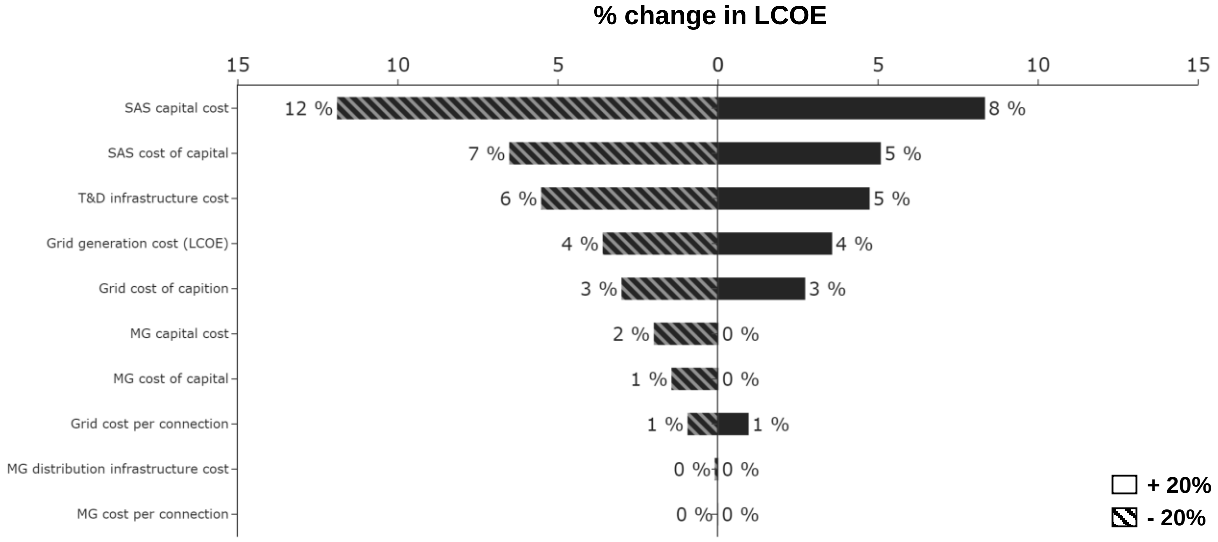


**Figure S5:** Sensitivity analysis for the reference scenario (Tier 3). Shows the percentage change in the weighted average LCOE for all of sub-Saharan Africa for a variation of ± 20% in each input parameter.

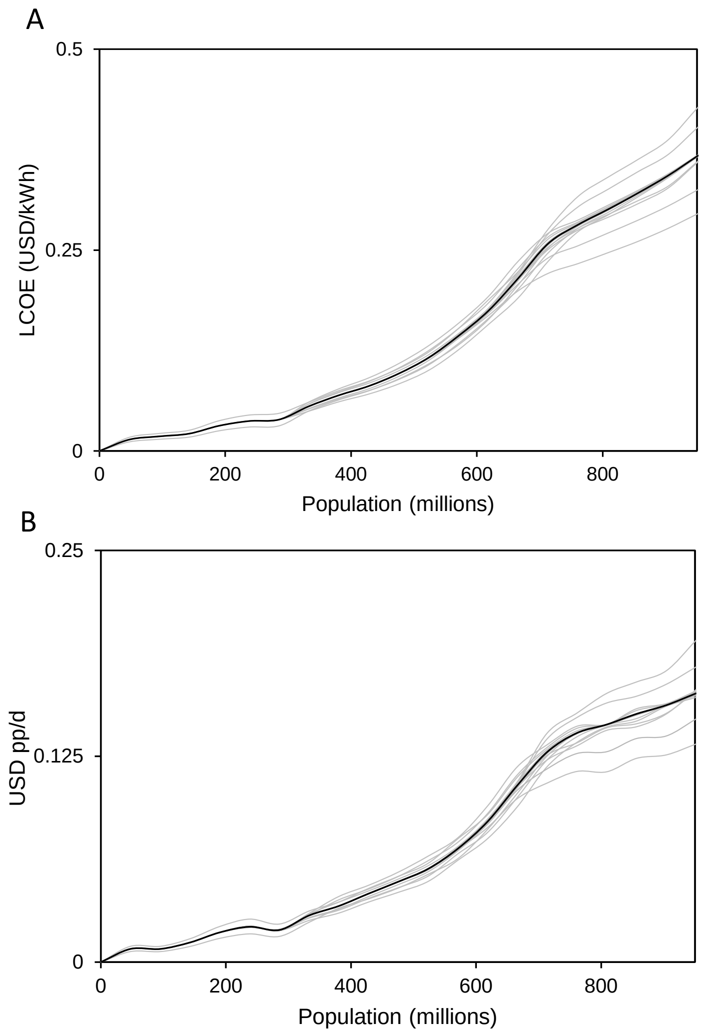


**Figure S6:** Sensitivities along the electrification cost curve. A. LCOE. B. Cost pp/d. Black line represents the baseline Tier 3 cost curve as shown in Figure 2 in the paper. Grey lines show the sensitivity for each ± 20% variation shown in Figure S4 (ten lines above and ten below the baseline).

## Additional Tables

**Table S1**: Description of demand tiers used in analysis[2]

| | Tier 2 | Tier 3 | Tier 4 |
|---|---|---|---|
| Indicative annual per household demand (kWh) | ca. 73 | ca. 365 | ca. 1,241 |
| Availability (minimum duration (hours)) | 4 – 8 hours | 8 -16 hours | 16 – 22 hours |
| Examples of typical energy services | Electrical lighting, phone charging & televisions | Tier 2 and mid-level powered appliances e.g., refrigerator/freezer, fans, water pump | Tier 3 highly powered appliances e.g., air conditioner/space heater, electric cooker, water heater |

**Table S2:** Off-grid electrification assumptions (based on OnSSET and Table S3, see methods)

| Parameter | CAPEX (USD/kW) | O&M (in % of CAPEX) | Asset lifetime (Years) | Capacity factor |
|---|---|---|---|---|
| Solar PV mini-grid (including batteries) | 2,139 | 1.5% + 2% of T&D | 20 | Calculated using solar resource availability data |
| Hydro mini-grid | 3,000 | 1.5% + 2% of T&D | 30 | 0.5 |
| Solar PV standalone system (including batteries) | <0.02 kW = 6,720<br>0.02 < kW < 0.05 = 6,133<br>0.05 < kW < 0.1 = 4,457<br>kW > 0.1 = 3,122 | 2% | 15 | Calculated using solar resource availability data |

**Table S3:** Annual cost reduction factors by component and system

| Parameter | Annual cost reduction factor |
|---|---|
| *Components* | |
| PV Modules | 5% |
| Li-ion Battery | 12% |
| Inverter, charge controllers & BOS | 5% |
| *System (total CAPEX)* | |
| Mini-grid | 4% |
| Standalone system | 5% |

**Table S4:** MG and SAS future cost triangulation

| Approach | Source | Region | Capital cost (USD/kW) | Year |
|---|---|---|---|---|
| Mini-grid | National Renewable Energy Lab[3] | Sub-Saharan Africa | 3,356 | 2020 |
| | UNDP Cambodia[4] | Cambodia | 2,566 | 2025 |
| | The World Bank/Energy Sector Management Assistance Program[5] | Global | 2,500 | 2030 (best in class) |
| Standalone system | Rockefeller Foundation[6] | Global, focus Sub-Saharan Africa | 7,580 | 2020 (0.05 kW system) |
| | UNDP Cambodia[4] | Cambodia | 3,360 | 2025 (0.1 kW system) |

**Table S5:** Cost increase due to misguided planning. The second column shows the population weighted average LCOE across SSA as shown in the paper (optimal electrification approach choice). The third column shows the population weighted average LCOE across SSA if the electrification approach is chosen according to the previous column but electrification demand is only Tier 2. Technically, we show the LCOE of the Tier 2 model run for the (often suboptimal) electrification approach from the first column. Note that OnSSET only calculates the grid LCOE if the optimal technology choice is grid. Hence, most clusters that are electrified via the grid in Tier 3 or Tier 4 but via and off grid approach in Tier 2 are excluded because we do not have a Tier 2 grid electrification cost. We report the according population coverage for both cases in the first column.

| | Share of population covered | Weighted average LCOE; expected demand (USD/kWh) | Weighted average LCOE; T2 demand (USD/kWh) | Relative increase in LCOE |
|---|---|---|---|---|
| Tier 3 | 81% | 0.13 | 0.17 | 32% |
| Tier 4 | 72% | 0.09 | 0.17 | 86% |

**Table S6:** Cost increase due to oversizing. We choose two representative clusters at the median of the Tier 3 LCOE distribution by electrification approach. Survey data from an electrification program evaluation in Rwanda show that newly grid connected households consumed 11 kWh/month on average (median = 6 kWh/month) and official data for the same country reporting that more than half of newly grid connected households consume less than 15 kWh/month[7]. 15 kWh/month is roughly half of the assumed Tier 3 demand in OnSSET (51% for the representative MG cluster, 53% for SAS respectively). We therefore contrast the LCOE for Tier 3 electrification with a case where demand is reduced to 50%. Note that Tier 3 demand is country specific.

| | Country specific Tier 3 demand per household (kWh/yr) | LCOE for Tier 3 demand (USD/kWh) | LCOE for 50% of Tier 3 demand (USD/kWh) | Relative increase in LCOE |
|---|---|---|---|---|
| Representative MG | 353 | 0.35 | 0.79 | 128% |
| Representative SAS | 339 | 0.31 | 0.68 | 121% |

**Table S7:** Table showing the LCOE, cost pp/d and population per country

| | | Tier 2 | | Tier 3 | | Tier 4 | |
|---|---|---|---|---|---|---|---|
| **Country** | **Population to be electrified** | **LCOE (USD/kWh)** | **cost pp/d** | **LCOE (USD/kWh)** | **cost pp/d** | **LCOE (USD/kWh)** | **cost pp/d** |
| Angola | 31,787,200 | 0.16 | 0.02 | 0.13 | 0.05 | 0.11 | 0.10 |
| Benin | 10,689,460 | 0.15 | 0.03 | 0.10 | 0.07 | 0.07 | 0.13 |
| Botswana | 1,345,063 | 0.09 | 0.03 | 0.08 | 0.09 | 0.07 | 0.20 |
| Burkina Faso | 22,444,490 | 0.26 | 0.03 | 0.25 | 0.11 | 0.23 | 0.28 |
| Burundi | 14,789,410 | 0.27 | 0.04 | 0.15 | 0.09 | 0.10 | 0.15 |
| Cameroon | 17,926,060 | 0.15 | 0.03 | 0.09 | 0.06 | 0.06 | 0.11 |
| Central African Republic | 4,702,520 | 0.31 | 0.04 | 0.29 | 0.12 | 0.27 | 0.29 |
| Chad | 19,919,067 | 0.29 | 0.03 | 0.26 | 0.11 | 0.24 | 0.26 |
| Democratic Republic of Congo | 104,877,598 | 0.22 | 0.03 | 0.15 | 0.07 | 0.12 | 0.15 |
| Republic of Congo | 3,754,890 | 0.21 | 0.03 | 0.19 | 0.11 | 0.16 | 0.25 |
| Equatorial Guinea | 990,840 | 0.16 | 0.02 | 0.09 | 0.04 | 0.05 | 0.06 |
| Eritrea | 4,227,450 | 0.26 | 0.03 | 0.19 | 0.08 | 0.15 | 0.17 |
| Kingdom of Eswatini | 637,100 | 0.21 | 0.05 | 0.17 | 0.16 | 0.10 | 0.25 |
| Ethiopia | 93,697,777 | 0.17 | 0.02 | 0.13 | 0.06 | 0.09 | 0.11 |
| Gabon | 764,454 | 0.08 | 0.01 | 0.06 | 0.03 | 0.05 | 0.07 |
| Gambia | 1,788,830 | 0.21 | 0.02 | 0.18 | 0.06 | 0.16 | 0.13 |
| Ghana | 14,017,460 | 0.13 | 0.02 | 0.10 | 0.06 | 0.07 | 0.12 |
| Guinea | 13,062,830 | 0.28 | 0.03 | 0.21 | 0.08 | 0.17 | 0.16 |
| Guinea Bissau | 1,996,760 | 0.22 | 0.02 | 0.20 | 0.08 | 0.18 | 0.19 |
| Kenya | 34,351,350 | 0.13 | 0.02 | 0.10 | 0.07 | 0.07 | 0.13 |
| Lesotho | 1,838,540 | 0.22 | 0.05 | 0.13 | 0.10 | 0.08 | 0.16 |
| Liberia | 5,476,040 | 0.19 | 0.02 | 0.14 | 0.06 | 0.10 | 0.11 |
| Madagascar | 29,288,820 | 0.28 | 0.04 | 0.25 | 0.14 | 0.23 | 0.32 |
| Malawi | 24,086,800 | 0.23 | 0.04 | 0.14 | 0.08 | 0.10 | 0.16 |
| Mauritania | 4,198,292 | 0.26 | 0.02 | 0.18 | 0.06 | 0.14 | 0.12 |
| Mozambique | 34,195,870 | 0.26 | 0.04 | 0.20 | 0.11 | 0.15 | 0.21 |
| Namibia | 1,882,080 | 0.15 | 0.03 | 0.15 | 0.10 | 0.13 | 0.24 |
| Niger | 30,531,340 | 0.26 | 0.03 | 0.18 | 0.06 | 0.13 | 0.12 |
| Nigeria | 158,295,030 | 0.16 | 0.02 | 0.10 | 0.05 | 0.08 | 0.10 |
| Rwanda | 11,773,910 | 0.22 | 0.03 | 0.12 | 0.05 | 0.08 | 0.09 |
| Senegal | 12,020,950 | 0.19 | 0.01 | 0.15 | 0.04 | 0.12 | 0.09 |
| Somalia | 16,313,682 | 0.30 | 0.03 | 0.29 | 0.10 | 0.26 | 0.25 |
| South Africa | 16,666,174 | 0.10 | 0.02 | 0.08 | 0.07 | 0.06 | 0.14 |
| South Sudan | 16,130,098 | 0.27 | 0.03 | 0.21 | 0.07 | 0.17 | 0.16 |
| Sudan | 33,740,812 | 0.30 | 0.03 | 0.26 | 0.09 | 0.23 | 0.23 |
| Tanzania | 64,202,020 | 0.17 | 0.02 | 0.13 | 0.07 | 0.09 | 0.13 |
| Togo | 6,640,742 | 0.16 | 0.03 | 0.10 | 0.06 | 0.07 | 0.12 |
| Uganda | 54,102,740 | 0.17 | 0.03 | 0.09 | 0.06 | 0.06 | 0.09 |
| Zambia | 17,815,780 | 0.22 | 0.03 | 0.19 | 0.08 | 0.15 | 0.18 |
| Zimbabwe | 14,761,660 | 0.26 | 0.04 | 0.22 | 0.12 | 0.16 | 0.23 |
| **Total / overall** | **951,731,988** | **0.20** | **0.03** | **0.14** | **0.07** | **0.11** | **0.15** |

**Table S8**: country specific inputs for households, grid costs and the cost of capital used for analysis.

| Country | Population 2030 | NumPeoplePerHH (Urban) | NumPeoplePerHH (Rural) | Grid Capacity Investment Cost (USD/kW) | Grid Losses | GridGenerationCost (USD/kWh) | Cost of capital | | |
|---|---|---|---|---|---|---|---|---|---|
| | | | | | | | Grid | Mini-grid | Standalone system |
| Angola | 44,427,789 | 5.6 | 6.7 | 1,160 | 0.11 | 0.019 | 8.1% | 21.2% | 15.3% |
| Benin | 15,648,264 | 3.1 | 3.6 | 1,810.7 | 0.24 | 0.031 | 7.6% | 21.0% | 14.9% |
| Botswana | 2,749,922 | 1.7 | 2.1 | 1,505.2 | 0.11 | 0.032 | 2.6% | 15.7% | 9.8% |
| Burkina Faso | 27,259,037 | 4.4 | 5.1 | 1,998 | 0.17 | 0.169 | 10.0% | 23.2% | 17.2% |
| Burundi | 15,783,471 | 3.5 | 4.1 | 973 | 0.24 | 0.033 | 13.2% | 26.4% | 20.4% |
| Cameroon | 3,283,193 | 3 | 3.3 | 1,980.1 | 0.11 | 0.017 | 9.1% | 22.3% | 16.3% |
| Central Africa Republic | 6,031,242 | 5 | 5.6 | 2,228 | 0.14 | 0.134 | 11.9% | 25.0% | 19.1% |
| Chad | 21,450,533 | 5.1 | 5.6 | 1,924.1 | 0.31 | 0.108 | 11.2% | 24.3% | 18.4% |
| Republic of Congo | 7,262,400 | 3.5 | 3.9 | 1,782 | 0.45 | 0.034 | 10.6% | 23.8% | 17.8% |
| Democratic Republic of Congo | 119,763,914 | 4.4 | 4.9 | 1,713 | 0.21 | 0.02 | 10.5% | 23.7% | 17.7% |
| Equatorial Guinea | 1,853,489 | 5.2 | 5.7 | 2,188 | 0.25 | 0.02 | 9.8% | 23.2% | 17.1% |
| Eritrea | 6,751,715 | 4.5 | 5.4 | 1,448.4 | 0.13 | 0.06 | 14.7% | 27.9% | 21.9% |
| Eswatini | 1,673,865 | 2.2 | 2.6 | 1,693.1 | 0.61 | 0.032 | 8.4% | 21.3% | 15.5% |
| Ethiopia | 139,578,721 | 4.4 | 5.2 | 2,248 | 0.19 | 0.018 | 7.7% | 20.9% | 14.9% |
| Gabon | 2,632,143 | 4 | 4.4 | 2,184.4 | 0.28 | 0.024 | 8.8% | 21.9% | 16.0% |
| Gambia | 3,028,782 | 6.6 | 7.6 | 1,207.5 | 0.4 | 0.062 | 9.8% | 23.3% | 17.2% |
| Ghana | 37,413,133 | 3.3 | 3.8 | 1,801 | 0.23 | 0.03 | 10.2% | 23.4% | 17.4% |
| Guinea | 17,618,044 | 5.6 | 6.5 | 1,985.8 | 0.58 | 0.015 | 14.6% | 28.3% | 22.1% |
| Guinea-Bissau | 2,466,973 | 5 | 5.8 | 1,307.8 | 0.4 | 0.137 | 8.8% | 22.3% | 16.2% |
| Kenya | 67,000,459 | 3 | 3.5 | 1,906 | 0.18 | 0.022 | 8.0% | 21.2% | 15.2% |
| Lesotho | 2,653,958 | 2.6 | 3.1 | 2,181.1 | 0.22 | 0.026 | 10.6% | 24.0% | 17.9% |
| Liberia | 6,513,217 | 4.6 | 5.4 | 2,180.8 | 0.25 | 0.012 | 14.2% | 27.8% | 21.6% |
| Madagascar | 35,370,576 | 3.7 | 4.4 | 2,353 | 0.33 | 0.1355 | 8.2% | 21.6% | 15.5% |
| Malawi | 26,433,006 | 3.5 | 4.1 | 1,362.9 | 0.23 | 0.047 | 11.3% | 24.9% | 18.7% |
| Mauritania | 5,981,526 | 6.1 | 7 | 1,628 | 0.27 | 0.042 | 15.1% | 28.5% | 22.4% |
| Mozambique | 41,989,931 | 3.7 | 4.4 | 1,737 | 0.15 | 0.017 | 13.0% | 26.2% | 20.2% |
| Namibia | 3,258,844 | 2.9 | 3.4 | 1,067.3 | 0.36 | 0.038 | 4.6% | 17.8% | 11.8% |
| Niger | 34,887,762 | 5.7 | 6.6 | 1,785 | 0.42 | 0.039 | 14.2% | 27.8% | 21.6% |
| Nigeria | 263,463,721 | 4 | 4.6 | 1,864.3 | 0.16 | 0.039 | 8.0% | 21.1% | 15.2% |
| Rwanda | 16,040,538 | 4.7 | 5.6 | 1,240.5 | 0.19 | 0.042 | 8.8% | 22.0% | 16.0% |
| Senegal | 22,179,714 | 7.2 | 8.4 | 1,303.4 | 0.13 | 0.066 | 7.3% | 20.4% | 14.5% |
| Somalia | 21,420,425 | 5.6 | 6.6 | 1,759 | 0.25 | 0.155 | 15.5% | 29.2% | 23.0% |
| South Africa | 64,679,757 | 2.3 | 2.7 | 2,356.9 | 0.08 | 0.036 | 4.2% | 17.4% | 11.4% |
| South Sudan | 17,147,041 | 5.7 | 6.8 | 2,941.1 | 0.06 | 0.036 | 10.5% | 24.2% | 18.0% |
| Sudan | 54,519,982 | 5.5 | 6.5 | 1,183 | 0.14 | 0.036 | 18.5% | 32.2% | 26.0% |
| Tanzania | 83,285,995 | 3.9 | 4.6 | 1,345.5 | 0.18 | 0.032 | 8.4% | 21.9% | 15.8% |
| Togo | 10,509,876 | 3.3 | 3.8 | 2,020.4 | 0.37 | 0.03 | 9.7% | 23.2% | 17.1% |
| Uganda | 63,901,008 | 3.4 | 4 | 2,300 | 0.17 | 0.012 | 8.5% | 21.7% | 15.7% |
| Zambia | 24,802,597 | 4.5 | 5.3 | 1,441.1 | 0.15 | 0.013 | 10.2% | 23.4% | 17.4% |
| Zimbabwe | 21,433,286 | 3.7 | 4.3 | 1,884.3 | 0.16 | 0.025 | 12.3% | 25.9% | 19.7% |

**Supplementary information references**


1. United Nations. *World Population Prospects*. https://population.un.org/wpp/ (2022).
2. Bhatia, M. & Angelou, N. *Beyond Connections - Energy Access Redefined*. (2015).
3. NREL. *U.S. Solar Photovoltaic System and Energy Storage Cost Benchmark: Q1 2020*. (2021).
4. UNDP. *Cambodia: De-risking Renewable Energy Investment*. (2019).
5. ESMAP. *Mini Grids for Half a Billion People: Market Outlook and Handbook for Decision Makers*. (2022).
6. Rockefeller Foundation. *Electrifying Economies: Detailed cost models and benchmarks*. (2022).
7. Lenz, L., Munyehirwe, A., Peters, J. & Sievert, M. Does Large-Scale Infrastructure Investment Alleviate Poverty? Impacts of Rwanda's Electricity Access Roll-Out Program. *World Dev.* **89**, 88–110 (2017).
